# Identification of sequence polymorphism in the D-Loop region of mitochondrial DNA as a risk factor for hepatocellular carcinoma with distinct etiology

**DOI:** 10.1186/1756-9966-29-130

**Published:** 2010-09-18

**Authors:** Ruixing Zhang, Fengbin Zhang, Cuiju Wang, Shunxiang Wang, Yih-Horng Shiao, Zhanjun Guo

**Affiliations:** 1 Department of Gastroenterology and Hepatology, The Fourth Hospital of Hebei Medical University, Shijiazhuang, PR China; 2 Department of Gynecology Ultrasound, The Fourth Hospital of Hebei Medical University, Shijiazhuang, PR China; 3 Department of Hepatobiliary Surgery, The Fourth Hospital of Hebei Medical University, Shijiazhuang, PR China; 4 Laboratory of Comparative Carcinogenesis, National Cancer Institute at Frederick, Frederick, MD 21702, USA

## Abstract

**Background:**

Hepatocellular carcinoma (HCC) is frequently preceded by hepatitis virus infection or alcohol abuse. Genetic backgrounds may increase susceptibility to HCC from these exposures.

**Methods:**

Mitochondrial DNA (mtDNA) of peripheral blood, tumor, and/or adjacent non-tumor tissue from 49 hepatitis B virus-related and 11 alcohol-related HCC patients, and from 38 controls without HCC were examined for single nucleotide polymorphisms (SNPs) and mutations in the D-Loop region.

**Results:**

Single nucleotide polymorphisms (SNPs) in the D-loop region of mt DNA were examined in HCC patients. Individual SNPs, namely the 16266C/T, 16293A/G, 16299A/G, 16303G/A, 242C/T, 368A/G, and 462C/T minor alleles, were associated with increased risk for alcohol- HCC, and the 523A/del was associated with increased risks of both HCC types. The mitochondrial haplotypes under the M haplogroup with a defining 489C polymorphism were detected in 27 (55.1%) of HBV-HCCand 8 (72.7%) of alcohol- HCC patients, and in 15 (39.5%) of controls. Frequencies of the 489T/152T, 489T/523A, and 489T/525C haplotypes were significantly reduced in HBV-HCC patients compared with controls. In contrast, the haplotypes of 489C with 152T, 249A, 309C, 523Del, or 525Del associated significantly with increase of alcohol-HCC risk. Mutations in the D-Loop region were detected in 5 adjacent non-tumor tissues and increased in cancer stage (21 of 49 HBV-HCC and 4 of 11 alcohol- HCC, p < 0.002).

**Conclusions:**

In sum, mitochondrial haplotypes may differentially predispose patients to HBV-HCC and alcohol-HCC. Mutations of the mitochondrial D-Loop sequence may relate to HCC development.

## Background

Hepatocellular carcinoma (HCC) is the fifth most frequent cancer and the third leading cause of cancer death worldwide, with over a half million mortality every year [1]. HCC is also common in China. The recent report for annual incidence and mortality in China were 300,000 and 306,000 cases [2,3]. This disease is strongly associated with several risk factors, including chronic hepatitis B virus (HBV) and chronic hepatitis C virus (HCV) infection, and alcohol abuse [4]. HBV infection is a challenging health issue in China, where about 93 million peoples are HBV carriers and 30 million have chronic B hepatitis [5]. Alcohol abuse is also on the rise in China and about 6.6% of males and 0.1% of females are diagnosed with alcohol dependence [6]. Many of these patients develop liver diseases, such as alcoholic hepatitis and cirrhosis, which are prone to HCC.

Hepatitis virus infection and alcohol abuse are associated with increased oxidative stress in liver cells, resulting in DNA changes including mitochondrial DNA (mtDNA) instability [7,8]. The human mitochondrial genome is 16 kb in length and a closed-circular duplex molecule that contains 37 genes, including two ribosomal RNAs and complete set of 22 tRNAs [9]. mtDNA is believed to be more susceptible to DNA damage and acquires mutations at a higher rate than nuclear DNA because of high levels of reactive oxygen species (ROS), lack of protective histones, and limited capacity for DNA repair in mitochondria [10-12]. Thus, somatic mtDNA mutations occur in a wide variety of degenerative diseases and cancers [13,14], and can be homoplasmic by clonal expansion [15,16] or heteroplasmic in tumor tissues [17,18]. In many cancers, including hepatitis virus-related HCC, somatic mutations are frequently located in mtDNA noncoding region called D-Loop [19,20]. This region is important for regulating both replication and expression of the mitochondrial genome because it contains the leading-strand origin of replication and the main promoter for transcription [21]. Ethanol also increases ROS generation in hepatic mitochondria and is capable of inducing multiple hepatic mitochondrial DNA deletions [8,22]. Somatic mutations in mitochondria have been rarely studied in alcohol-related HCC patients.

Sequence changes have been examined extensively in the D-Loop in cancers [17,19,20], but it is not clear whether those changes represent real somatic mutations or single nucleotide polymorphisms (SNPs), because blood mitochondria DNAs were not analyzed. Although some studies focus on sequence variant determination using blood DNA, only few SNPs have been selected for predicting cancer risk and their predictive values are still unclear [23-26]. The D-loop contains a length of 1122 bps (nucleotide 16024-16569 and 1-576) refers to mitochondria database http://www.mitomap.org In this study, we sequenced a region of about 1 kb franking almost all the D-Loop in cancerous and adjacent noncancerous tissues, and blood from the same patients of both hepatitis B virus-related (HBV-HCC) and alcohol-related HCC (alcohol-HCC). Many polymorphisms and somatic mutations were identified. When compared with controls without HCC, these genetic information are particular valuable to predict risk and to reveal natural history of the two types of HCC.

## Methods

### Tissue specimens and mtDNA extraction

We obtained histologically confirmed cancerous and corresponding noncancerous liver tissues from patients of 11 alcohol-HCC (average alcohol consumption higher than 40 g per day for at least five years) and 49 HBV- HCC, and liver tissues with no detectable malignancies except hepatic hemangioma from 38 control patients at the Fourth Hospital of Hebei Medical University. The hemangioma patients under surgery were selected as control just because it was vascular malformation with developmental aberration and we can obtain normal liver tissue from the specimen. Clinical characteristics of HCC patients and controls were listed in Table 1 and only one patient with alcohol abuse was found in the virus group. The liver function of all patients belonged to the Child-Pugh A or B cirrhosis index with total bilirubin levels less than 30 umol/L. No difference in tumor pathology could be found between alcohol-HCC and HBV-HCC. The HBV-HCC patients were apparently carriers for HBV. The histological specimens were independently reviewed by two pathologists. If initial examination did not agree, consensus was obtained after joint microscopic evaluation. All tissues were kept in liquid nitrogen immediately after surgical resection according to guideline of the human tissue research committee at the hospital, Written informed consent was obtained from all participants prior to enrollment. Mitochondria were isolated from liver tissue and mtDNA was extracted with the Mitochondrial DNA Extraction Kit (Genmed Scientific Inc, Shanghai, China). Whole blood was obtained from corresponding HCC patients and controls except in one case without an available blood sample in the alcohol-HCC group. Mitochondria isolation and mtDNA extraction were carried out using the Blood Mitochondrial DNA Extraction Kit (Genmed Scientific Inc.). All mtDNA was stored at -20°C.

**Table 1:** Clinical data in HBV-HCC, alcohol-HCC patients and controls

	HBV-HCC (n = 49)	Alcohol-HCC (n = 11)	Control (n = 38)
Age (years)	52.20 ± 9.86	58.36 ± 8.11	53.08 ± 10.98

Sex (M/F)	43/6	10/1	18/20

Child-Pugh Grade (B/A)	2/47	0/11	-

Alcohol abuse	1	11	0

Positive HBV surface antigen	49	0	0
Positive HBV anti-surface antibody	0	0	0
Tumor stage (I/II/III)	13/36/0	2/5/3^a^	-

^a^One alcohol-HCC patient did not have sufficient tissues for stage classification.

### PCR amplification and sequence analysis

The forward primer 5'-CCCCATGCTTACAAGCAAGT-3' (nucleotide 16190-16209) and reverse primer 5'-GCTTTGAGGAGGTAAGCTAC-3' (nucleotide 602-583) were used for amplification of a 982 bp product from mtDNA D-Loop region as described previously [27]. PCR was performed according to the protocol of PCR Master Mix Kit (Promega, Madison, WI) and purified prior to sequencing. Cycle sequencing was carried out with the Dye Terminator Cycle Sequencing Ready Reaction Kit (Applied Biosystem, Foster City, CA) and the products were then separated on the ABIPRISM Genetic Analyzer 3100 (Applied Biosystem). Mutations and polymorphisms were confirmed by repeated analyses from both strands. SNPs were identified directly from blood mitochondria.

### Statistical analysis

Paired and unpaired Student's t-test were used as appropriate to determine the differences SNP distribution within the D-Loop region and the number of SNPs per patient among groups. Fisher's exact test and chi-square were used accordingly to analyze dichotomous values, such as presence or absence of an individual SNP in each patient group. A p value of less than 0.05 was considered statistically significant. The Wilcoxon rank sum test was used to determine statistical differences among age, sex and Child-Pugh grade. Pairwise linkage disequilibrium between SNPs was done using GENEPOP http://wbiomed.curtin.edu.au/genepop

## Results

SNPs in reference to GenBank accession AC_000021 were detected in 92 sites of the 982-bp mitochondria D-Loop region from blood samples. The minor allele frequency ranged from 1.0% (1/98) to 46.90 (46/98). Of these, 13 SNPs (16A/T, 44C/CC, 56A/G, 245T/C, 275G/A, 310T/G, 368A/G, 449T/C, 454T/C, 570C/G, 16259C/G, 16267C/G, and 16445T/C) were new, as they were not reported in a mitochondria database http://www.mitomap.org. SNP numbers ranged from 3 to 13 for individuals, no statistical difference for SNP numbers in each individual referring to sex was observed. The pairwise linkage disequilibrium analysis between the SNPs identified in the D-loop of HCC patients and controls was performed, the paired loci with linkage disequilibrium p value less than 0.001 were listed(Additional File 1, Table S1).

**Figure 1:**
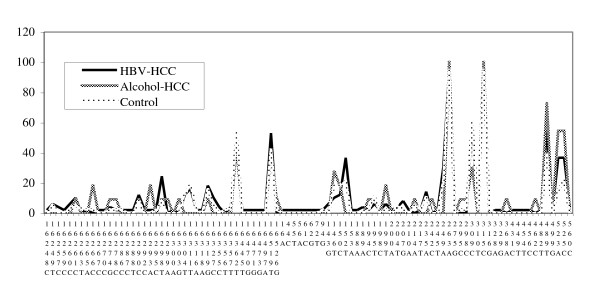
**Distribution (spectrum) of D-Loop SNPs at 92 sites (x-axis) and their relative frequencies in percentage within each group (y-axis)**. Paired T-test: p = 0.0001 (HBV-HCC vs. control); p = 0.3416 (Alcohol-HCC vs. control); p = 0.2817 (HBV-HCC vs. Alcohol-HCC).

HBV-HCC patients clearly showed a significantly higher SNP frequency referring to the numbers of SNPs identified in each individual than control patients (Table 2). A tendency toward an increased SNP frequency was also observed for alcohol-HCC patients but did not reach statistical significance. Next, distributions or spectra of relative frequencies across 92 SNP sites from blood of patients in the HBV-HCC, alcohol-HCC, and control groups were compared to provide the topology of polymorphisms (Fig. 1). The diversity of distribution was analyzed by paired t-test and SNPs in HBV-HCC patients apparently showed distinct spectrum from control (p = 0.0001). The SNP distribution in the D-Loop region in alcohol-HCC appeared to be less differentiable from HBV-HCC and control.

**Table 2:** Average SNP frequency in the mitochonrial DNA D-Loop for each group.

	Control (n = 38)	HBV-HCC (n = 49)	Alcohol-HCC (n = 10)
SNPs/patient	6.7 ± 2.0^b^	8.5 ± 2.2	8.0 ± 1.9

P value^a^		0.0002	0.0730

^a^T test.

^b^Mean ± standard deviation

When individual SNPs were analyzed between HCC and control, a statistically significant increase of SNP frequency was observed for 16298C and 523del alleles in HBV-HCC (p < 0.05) and for 16293G, 523del, and 525del alleles in alcohol-HCC (p < 0.05) patients (Table 3). The trend was next determined with all 3 groups using t test. Additional SNPs (16266T, 16299G, 16303A, 242T, 368G, and 462T) were significantly associated with the tendency toward the increased risk for alcohol-HCC. In contrast, the 152C allele was correlated with reduced risk, especially for alcohol-HCC. The remaining 81 SNPs were not associated with either type of HCC.

**Table 3:** SNP sites showing frequency difference between HCC and control.

**Nucleotide**^**a**^	Control	HBV-HCC	Alcohol-HCC	**Trend-p value**^**b**^
16266 C/T	37/1 (2.6)^c^	49/0 (0.0)	8/2 (20.0)	**0.0038**^d^

P value		0.4368	0.1058	

16293 A/G	38/0 (0.0)	48/1 (2.0)	8/2 (20.0)	**0.0042**

P value		>0.9999	**0.0399**	

16298 T/C	35/3 (7.9)	37/12 (24.5)	9/1 (10.0)	0.0992

P value		**0.0495**	>0.9999	

16299A/G	38/0 (0.0)	49/0 (0.0)	9/1 (10.0)	**0.0123**

P value		>0.9999	0.2083	

16303 G/A	38/0 (0.0)	49/0 (0.0)	9/1 (10.0)	**0.0123**

P value		>0.9999	0.2083	

152 T/C	30/8 (21.1)	31/18 (36.7)	10/0 (0.0)	**0.0340**

P value		0.1130	0.1767	

242 C/T	38/0 (0.0)	49/0 (0.0)	9/1 (10.0)	**0.0123**

P value		>0.9999	0.2083	

368 A/G	38/0 (0.0)	49/0 (0.0)	9/1 (10.0)	**0.0123**

P value		>0.9999	0.2083	

462 C/T	38/0 (0.0)	49/0 (0.0)	9/1 (10.0)	**0.0123**

P value		>0.9999	0.2083	

523 A/del	32/6 (15.8)	31/18 (36.7)	4/6 (60.0)	**0.0122**

P value		**0.0302**	**0.0092**	

525C/del	30/8 (21.1)	31/18 (36.7)	4/6 (60.0)	**0.0483**

P value		0.1130	**0.0447**	

^a^Fisher's exact test: HCC vs. control.

^b^Chi-square test for trend.

^c^Number in parenthesis: SNP percentage.

^d^Number in bold: p < 0.05.

The M haplogroup, defined by the presence of 489C, was used to stratify the subject

groups for subsequent analysis. When the status of the 489C was combined with the above frequent SNPs, predictive values for the risks of HBV-HCC and alcohol-HCC were immediately detected in several haplotypes (Table 4). Frequencies of the 489T/152T, 489T/523A, and 489T/525C haplotypes were significantly reduced in HBV-HCC patients compared with controls. In contrast, the haplotypes of 489C with 152T, 249A, 309C, 523Del, or 525Del associated significantly with increase of alcohol-HCC risk. The haplotypes 489C/152T, 489C/523Del, and 489C/525Del further predicted the risk of alcohol-HCC in comparison with HBV-HCC. The other SNP-defined haplotypes did not associate with either type of HCC.

**Table 4:** Comparison of SNP frequencies with different 489 status among subject groups.

SNPs	Control (n = 38)	HBV-HCC (n = 49)	Alcohol-HCC (n = 11)	**P value**^**d**^
489T/152T	19 (50.0)^c^	13 (26.5)	3 (27.3)	>0.9999

P value		***0.0243***	0.3028	

489C/152T	11 (28.9)	18 (36.7)	8 (72.7)	**0.0437**

P value		*0.4447*	**0.0139**	

489C/249A	13 (34.2)	19 (38.8)	8 (72.7)	0.0513

P value		*0.6614*	**0.0372**	
489C/309C	6 (15.8)	12 (24.5)	6 (54.5)	0.0706

P value		*0.3204*	**0.0158**	

489T/523A	19 (50.0)	11 (22.4)	3 (27.3)	0.7075

P value		***0.0073***	0.3028	

489C/523Del	2 (5.3)	6 (12.2)	6 (54.5)	**0.0051**

P value		0.4571	**0.0007**	

489T/525C	18 (47.4)	10 (20.4)	3 (27.3)	0.6899

P value		***0.0076***	0.3106	

489C/525Del	3 (7.9)	6 (12.2)	6 (54.5)	**0.0051**

P value		0.7256	**0.0020**	

^a^HCC vs. control (Number/patient: unpaired T test; SNP-defined haplotypes: Fisher's Exact test, otherwise chi-square analysis to obtain values in italic).

^b^Mean ± standard deviation.

^c^Number in parenthesis: percentage.

^d^HBV-HCC vs. Alcohol-HCC.

In addition to SNPs, mutations in the D-Loop region were identified by comparing the sequences in tumor and adjacent non-tumor areas with the genotype in blood of the same subject, except for patient #1 whose blood DNA was not available for sequence analysis (Table 5). Instead, sequences from tumor and non-tumor tissues were compared for this patient. Mutations were detected in 21 of 49 HBV-HCC and in 4 of 11 alcohol-HCC patients. For 38 controls, identical D-Loop sequences were seen between blood and liver mtDNA of the same patient, confirming no mutations in liver tissues separated from hemangiomas. When statistical analysis was carried out using 38 controls as reference, significant increase of mutation frequency was observed in both HBV-HCC (Fisher's exact test, p = 0.0001) and alcohol-HCC (Fisher's exact test, p = 0.0016). Four patients, #18, #27, #60, and #65, in HBV-HCC and one patient, #14, in alcohol-HCC had mutations in non-tumor areas. These early mutations were localized at the same 309 site with either deletion or insertion of C.

**Table 5:** Mutation sites in cancer and adjacent noncancerous tissues

Patient	Blood	Noncancer	Cancer
HBV-HCC			
#4	309CC	309CC	309C
#6	16261C, 309CC	16261C,309CC	16261T, 309C
#8	309C	309C	309CC
#10	309CC	309CC	309C
#18	294T	294C	294C
#21	309CCC	309CCC	309CC
#22	309CC	309CC	309C
#24	72T	72T	72C
#26	309CC	309CC	309C
#27	51T, 309CCCC	51T, 309CCC	51C, 309CC
#29	Same as reference	Same as reference	313-320del
#34	189A	189A	189G
#40	60T	60T	60C
#42	309CCC	309CCC	309CC
#53	94G	94G	94A
#57	70G	70G	70A
#59	309CC	309CC	309C
#60	309CCC	309CC	309CC
#64	309CC	309CC	309C
#65	309CC	309C	309C
#66	309CCC	309CCC	309CC

Alcohol-HCC			
#1^a^	-	309CCC	309CC
#14	309CCC	309CC	309CC
#15	309CC	309CC	309C
#30	318T	318T	318C

^a^No blood mtDNA available.

## Discussion

An increase of mutations in the D-Loop region of mitochondria has been reported in HCC [19,20,27]. To predict cancer risk, selected SNPs in the D-Loop region have been examined in other tumor types [23-26]. The current study has extended those analyses to determine SNPs and mutations in a continuous sequence of mitochondrial DNA between nucleotides 16190 and 583 in patients of HCCs with different etiology, namely, HBV or alcohol abuse. This provides an opportunity to discover new SNPs and demonstrates that analysis of blood DNA along with tumor materials from the same patient is surely critical to differentiate SNPs from mutations.

SNPs appear to be common in this Chinese population with average of 7 to 9 for each patient in reference to GenBank AC_000021 sequence for Caucasians. The actual number of SNPs may be less if the reference sequence was of Chinese origin. These SNPs are less likely to arise from mutations in blood mitochondria DNA because the same SNPs were observed in corresponding non-tumor tissues. Also, they are homoplasmy with single peak detected at each SNP site. This suggests that the SNPs are germline sequence variants and also raises the possibility that some of homoplasmic mutations may actually have been SNPs in previous studies that do not have blood DNA for comparison. When compared with control, frequent SNPs in both HBV-HCC and alcohol-HCC patients provide the first evidence that a high SNP frequency seem to predisposes patients to HCC regardless of different etiology (Table 2). It is still unclear how SNPs in the D-loop transcription-regulatory region increase the risk of cancers, although these genetic changes have been frequently detected in many cancer types. There is evidence that production of ROS is enhanced when the mitochondrial transcription is altered [28]. This ROS-mediated mechanism may promote tumor formation. The spectrum across 92 SNP sites further shows a diverse pattern of SNPs in HBV-HCC patients compared with control (Fig. 1). The diversity was not prominent for alcohol-HCC, most likely due to small sample size. A new study is required to recruit more patients to examine the role of mtDNA D-Loop SNP frequency in alcohol-HCC risk.

From the SNP spectrum (Fig. 1), it is very easy to spot potential SNP sites showing either increase or decrease of frequency. In most SNP sites, the patterns of SNP distribution among HBV-HCC, alcohol-HCC, and control are very much overlapping each other. The weight for the sequence diversity appears to fall on the 16298T/C and 523A/del two SNPs for HBV-HCC, and 16293G/A, 523A/del, and 525C/del 3 SNPs for alcohol-HCC (Table 3). Several rare alleles defined as being less than 5% of allele frequency, though required confirmation in a larger population, tend to predict the risk of alcohol-HCC. These SNPs may be of great potentials for future studies of their biological functions. The predictive values of haplotypes, defined by combinations of the M haplogroup status with non-diagnostic but frequent SNPs, for the risks of HBV-HCC and alcohol-HCC are very provocative. The current study provides the evidence that these frequent SNPs nested within selected haplogroup may become useful predictors for cancer risk.

Mutations in the D-Loop region are also frequent in HBV-HCC and the frequency of 21/49 (42.9%, Table 5) is comparable to a report (39.3%) from another Chinese population [25]. The alcohol-HCC group appears to have a similarly high mutation frequency (4/11, 36.4%). The 309C/ins or 309C/del is still the most common type of mutation, as seen by others in many types of tumors [20,27]. Seventeen of the 60 HCC patients harbored somatic deletions/insertions at this mononucleotide repeat. The 309 repeat is part of the CSBII, which contributes to the formation of a persistent RNA-DNA hybrid to initiate the mtDNA replication [20,29,30], Some severe alteration in this repeat could lead to functional impairment of mitochondria and promote a growth advantage for tumor cell. Base changes persistent from adjacent noncancerous to cancerous areas in 4 of 21 HBV-HCC and 1 of 4 alcohol-HCC patients with mutations suggest that sequence alteration may occur early and may play a role in tumorigenesis. Mutation in adjacent non-tumor tissue with normal morphology, also observed by others [17,19], does not appear to be an incidental finding.

Although the mechanism of mutation is still unclear, free radicals generated in mitochondria could be responsible at least partly for these mutations. The D-loop region of mtDNA is important for regulation of mitochondrial genome replication and expression. Mutation in this region may affect mtDNA replication and may alter electron transport chain. All of these might contribute to early stage of hepatocarcinogenesis. Our data demonstrated that the utility of SNPs and mutations in mitochondria D-Loop region to predict HCC risk and to differentiate HCCs with distinct etiology. The utility of mtDNA SNPs for prediction of HCC risks from different environmental exposures is a promising area for future cancer prevention. Our results links genetic variation of exonuclear genome with cancer risk, subsequent research for SNP in D-loop is more difficult to perform than SNP in individual genes which can be compared by expressional levels, protein property and interacted genes. Future experiments with a large sample size are needed to explore the usage of those minor alleles and to validate the predictive values of SNPs identified in this pilot study.

## Competing interests

The authors declare that they have no competing interests.

## Authors' contributions

RZ and FZ contributed to experimental design, data acquisition and analyses. CW and SW contributed to experimental design, specimen collection, and data acquisition. YHS participated in data analyses, interpretation of results, and preparation of the manuscript. ZG contributed to conception, experimental design, data acquisition, analyses, and interpretation, and manuscript preparation. All authors read and approved the final manuscript.

## Supplementary Material

Additional file 1 **Table S1: Statitical significance of the pairwise linkage disequilibrium analysis among SNP in mitochondrial D-loop**. Click here for file
