# Supplementary material for: Identification of sequence polymorphism in the D-Loop region of mitochondrial DNA as a risk factor for hepatocellular carcinoma with distinct etiology
Source: J Exp Clin Cancer Res. 2010 Sep 18;29(1):130. doi: 10.1186/1756-9966-29-130 (PMC2949825; doi:10.1186/1756-9966-29-130)
Supplement: Additional file 1 — Table S1: Statitical significance of the pairwise linkage disequilibrium analysis among SNP in mitochondrial D-loop. [file 1756-9966-29-130-S1.DOC]

Table S1. Statitical significance of the pairwise linkage disequilibrium analysis among SNP in mitochondrial D-loop

| Locus pair *P*-Value |
| --- |
| 16298 vs 16327 0.000028 |
| 16290 vs 152 0.000357 |
| 16290 vs 525 0.000089 |
| 16304 vs 249 0.000002 |
| 16327 vs 249 0.000739 |
| 16362 vs 249 0.000007 |
| 16319 vs 523 0.000006 |
| 235 vs 525 0.000032 |
